# Interaction of the Main Components from the Traditional Chinese Drug Pair Chaihu-Shaoyao Based on Rat Intestinal Absorption

**DOI:** 10.3390/molecules16119600

**Published:** 2011-11-17

**Authors:** Yan Chen, Jinyan Wang, Ling Yuan, Lei Zhou, Xiaobin Jia, Xiaobin Tan

**Affiliations:** Key Laboratory of New Drug Delivery System of Chinese Material Medica, Jiangsu Province, Academy of Chinese Medicine, 100 Shizi Road, Nanjing 210028, China; Email: wwind924@yahoo.com.cn (J.W.); yuanl.china@sina.com (L.Y.); youleiwuheng@yahoo.com.cn (L.Z.); jxiaobin2005@hotmail.com (X.J.); jakytan2005@hotmail.com (X.T.)

**Keywords:** saikosaponin a, saikosaponin d, paeoniflorin, rat intestinal perfusion model, interaction

## Abstract

The Chaihu-Shaoyao drug pair (Bupleuri Radix and Paeoniae Radix Alba) which is a traditional Chinese drug pair, has been widely used for anti-inflammatory purposes. Saikosaponin a (SSA), saikosaponin d (SSD) and paeoniflorin are identified as the main components in the pair. The present study focused on the interaction of the main components based on investigating their intestinal absorption using a four-site perfused rat intestinal model in order to clarify the mechanism of the compatibility of Chaihu-Shaoyao. The concentrations of SSA, SSD and paeoniflorin in the intestinal perfusate were determined by LC/MS or UPLC (Ultra Performance Liquid Chromatography) methods, followed by P*_eff_ (effective permeability) and 10% ABS (the percent absorption of 10 cm of intestine) calculations. The results showed that all of the three main components displayed very low permeabilities (P*_eff_ < 0.4), which implied their poor absorption in the rat intestine. The absorption levels of SSA and SSD were similar in intestine and higher in ileum than those in other intestinal regions in the decreasing order: colon, jejunum and duodenum. However, there is no significant difference in the absorption of paeoniflorin in the four segments (P < 0.05). The P*_eff_ values of paeoniflorin exhibited an almost 2.11-fold or 1.90-fold increase in ileum when it was co-administrated with SSA and SSD, as well as 2.42-, 2.18-fold increase in colon, respectively, whereas the absorptions of SSA and SSD were not influenced by paeoniflorin. In conclusion, SSA and SSD could promote the absorption of paeoniflorin. To some extent this might explain the nature of the compatibility mechanisms of composite formulae in TCMs.

## 1. Introduction

Traditional Chinese Medicines (TCMs) have had a long therapeutic history for thousands of years [1], and composite formulae are one of the most important characteristics of TCMs to obtain synergistic effects or to diminish possible adverse reactions [2,3,4]. However, due to the complexity of the multi-components in TCMs, the compatibility mechanisms of most composite formulae have not been clearly elucidated. It is well known that oral administration is the main route for TCMs, and they should be absorbed in the gastrointestinal tract before they exhibit pharmacologic effects [5]. However, active components combined from different herbs might interact with each other in the gastrointestinal tract which could subsequently influence their absorption and their bioavailability. Therefore, the compatibility mechanisms of some composite formulae of TCMs might be clarified to some extent by the view of interaction of the active components based on their intestinal absorption.

**Figure 1:**
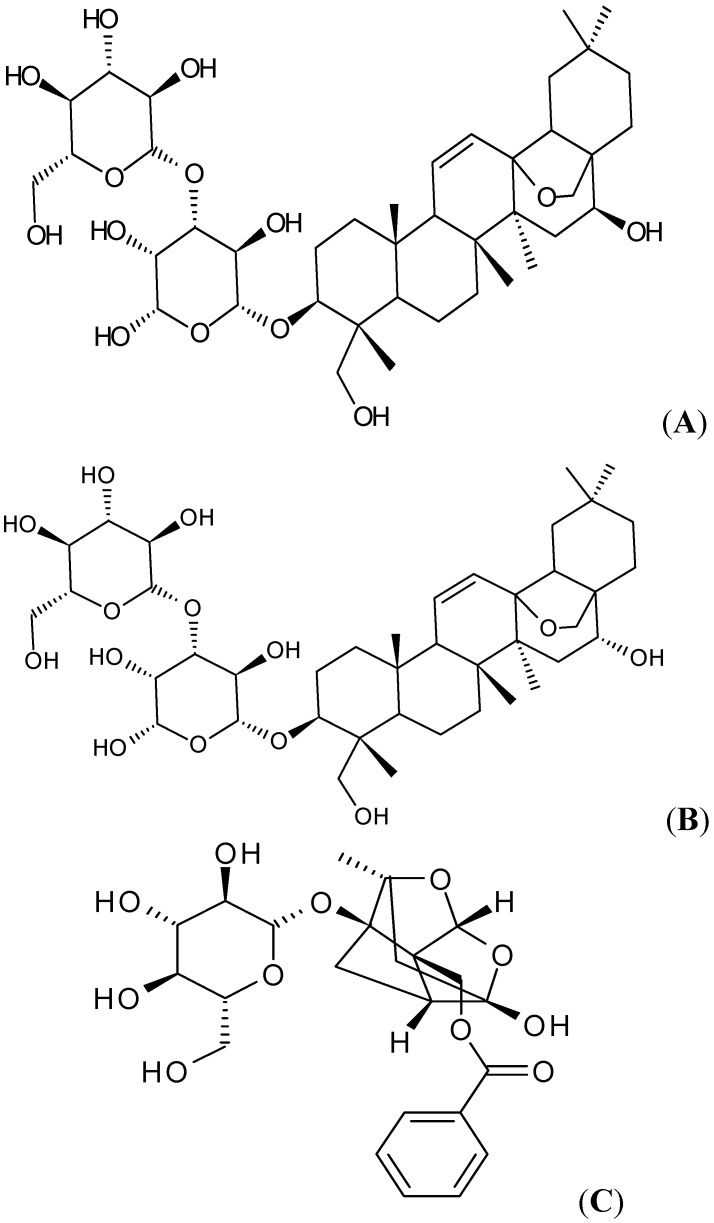
Chemical structures of SSA (**A**) SSD (**B**) and paeoniflorin (**C**).

Si-Ni-San is a famous traditional Chinese prescription found in the Treatise on Febrile Diseases of Zhang Zhongjing and has been widely used for thousands of years in treating various inflammatory diseases including gastritis, colitis and hepatitis [6,7]. It consists of four herbs, which are Shaoyao (Paeoniae Radix Alba), Chaihu (Bupleuri Radix), Zhishi (Aurantii Fructus Immaturus) and Gancao (Glycyrrhizae radix et rhizoma), and the Chaihu-Shaoyao drug pair is considered a classical drug pair in Si-Ni-San [8]. The main bio-active compounds in Chaihu are the saikosaponins, of which there are four types: saikosaponin a (SSA), b, c and d (SSD). The contents of saikosaponin b and saikosaponin c are pretty low in the raw material, and it is reported that SSA and SSD play a key role in the activity [9]. It is also reported that SSD has many pharmacological effects such as sedative action, anti-inflammatory, anti-tumor and anti-virus properties [10]. The main components in Shaoyao are monoterpenes and monoterpene glycosides, among which paeoniflorin, a monoterpene glycoside, is the most important component [8]. It also has been demonstrated that paeoniflorin has prominently sedative, analgesic, anti-inflammatory and two-way immunomodulatory activities [11,12,13]. Additionally, SSA, SSD and paeoniflorin are used as marker compounds to control the quality of Chaihu and Shaoyao in the Chinese Pharmacopoeia, respectively. (The chemical structures of SSA, SSD and paeoniflorin are shown in Figure 1).

So far the compatibility mechanism of Chaihu-Shaoyao drug pair has not been reported, nor has any study of the absorption of saikosaponins in Chaihu been reported. Therefore, our study focused on the intestinal absorption and interactions of the main components in the Chaihu-Shaoyao drug pair. In this paper, we examined the interaction of SSA (SSD) and paeoniflorin in order to clarify their compatibility mechanism. The traditional purpose of combining these two herbs together is for producing synergistic effect. Our findings indicated that saikosaponins could significantly improve the absorption of paenoiflorin, which to some extent provides the scientific evidence to support the synergistic effects of the two herbs. Here, the absorption and interactions of the main components in Chaihu-Shaoyao drug pair were first studied by using the rat intestinal perfusion model, which is routinely used to investigate drug absorption and metabolism and is recognized by the FDA as a viable model of human intestinal absorption [14,15].

## 2. Results and Discussion

### 2.1. Intestinal Absorption of SSA, SSD and Paeoniflorin

The absorptions of SSA, SSD and paeoniflorin in different intestinal segments were studied by the rat intestinal perfusion model. The concentrations of all the compounds of interest were 20 μmol·L^−1^. The concentrations of the compounds in the perfusate samples were analyzed by UPLC or LC/MS, and then the P*_eff_ and 10% ABS of SSA, SSD and paeoniflorin were calculated. 

The data presented in Table 1 showed that the P*_eff_ of SSA ranged from 0.193 to 0.207 and for SSD it ranged from 0.197 to 0.364 in the four intestinal segments, respectively. The low P*_eff_ (P*_eff_ < 1) of SSA and SSD suggested that SSA and SSD had a very poor absorption in the intestine. Although the P*_eff_ of SSD was slightly higher than that of SSA in the four intestinal segments, there was no significant differences from each other. The results showed that the absorption levels of SSA and SSD in the four segments were similar, and can be arranged in the following descending order: Ileum > colon > jejunum > duodenum, and the absorptions of these two compounds in the ileum were higher than that of other segments (P < 0.05), especially compared with that of duodenum, the P*_eff_ of ileum were approximately 2-fold higher than that of duodenum. The reason might be that ileum was where their selective absorption ocurred.

**Table 1:** Permeability of SSA, SSD and paeoniflorin in different intestinal segments (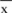 ± s, n = 4).

Compound	P*_eff_			
	Duodenum	Jejunum	Ileum	Colon
SSA	0.193 ± 0.028	0.207 ± 0.012	0.361 ± 0.014 *	0.239 ± 0.042
SSD	0.197 ± 0.036	0.248 ± 0.022	0.364 ± 0.055 *	0.337 ± 0.084
Paeoniflorin	0.268 ± 0.042	0.243 ± 0.030	0.214 ± 0.021	0.219 ± 0.051

The asterisk (*) indicates statistically significant differences in ileum from duodenum, jejunum (*P* < 0.05).

In addition, like SSA and SSD, the P*_eff_ of paeoniflorin was also pretty low. From Table 1 we can see that the result of paeoniflorin revealed that its rank order of permeability was duodenum (highest, P*_eff_ = 0.268), followed by jejunum (P*_eff_ = 0.243), colon (P*_eff_ = 0.219) and ileum (P*_eff_ = 0.214), whereas the absorption of paeoniflorin had no significant difference in the four intestinal segments. As summarized in Table 2, the tested ranges of the 10% ABS in the four segments were 3.75 to 6.26 for SSA, 4.52 to 6.10 for SSD and 5.23 to 6.57 for paeoniflorin. The above 10% ABS results were consistent with the P*_eff_ results. Meanwhile, no metabolite of these three compounds was found under the experimental conditions used in this study.

**Table 2:** 10% ABS of SSA, SSD and paeoniflorin in different intestinal segments (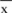 ± s, n = 4).

Compound	10 cm % ABS			
	Duodenum	Jejunum	Ileum	Colon
SSA	3.75 ± 0.57	3.91 ± 0.60	6.26 ± 0.56 *	3.84 ± 0.10
SSD	4.52 ± 0.77	4.69 ± 0.81	6.10 ± 0.34 *	5.35 ± 1.04
Paeoniflorin	6.57 ± 0.98	5.94 ± 0.70	5.23 ± 0.49	5.37 ± 1.19

The asterisk (*) indicates statistically significant differences in ileum from duodenum, jejunum (*P* < 0.05).

We employed LC/MS to analyze SSA and SSD in the perfusate, because their ultraviolet absorptions were not sensitive that it was difficult to detect them by UV or DAD [16]. Moreover, due to the strong ultraviolet absorption of paeoniflorin, UPLC with UV was used for analyze paeoniflorin in the perfusate and the method we developed was sensitive, rapid and reliability.

Taken together, the absorptions of SSA, SSD and paeoniflorin were poor in the intestine, even though they could be absorbed in the four intestinal segments. Their low permeabilities approximated 0.3, which were similar to that of mannitol, one of the typical compounds with poor absorption [17]. This might relate to the chemical structures of these three compounds. Their aglycones are connected with sugar moieties, and some literatures have reported that the presence of sugar moieties could affect solubility and permeability, which might lead to the low intrinsic permeability of compounds in the intestine [18]. In addition, SSA and SSD are a pair of stereoisomers, which have the same glucose-fucose disaccharide and molecular weights of 780, thereby their absorptions were similar in the intestine [19].

### 2.2. Interaction Between SSA, SSD and Paeoniflorin

#### 2.2.1. Effect of SSA or SSD on the Absorption of Paeoniflorin

**Figure 2:**
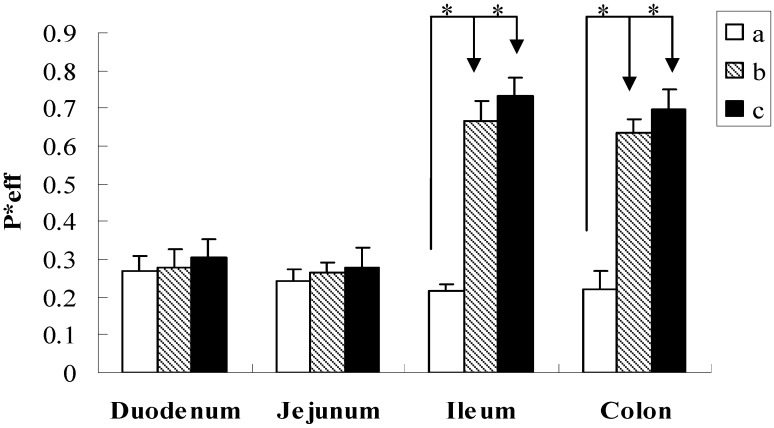
Effective permeability of paeoniflorin in different intestinal segments. (**a**) paeoniflorin alone; (**b**) paeoniflorin co-administration with SSA; (**c**) paeoniflorin co-administration with SSD. *P* < 0.05 *vs*. paeoniflorin alone.

The effect of SSA or SSD on the absorption of paeoniflorin was investigated and the results are depicted in Figure 2 and Table 2. When paeoniflorin was co-administrated with SSA (20:20 μmol·L^−1^), the P*_eff_ of paeoniflorin was 0.279 ± 0.049, 0.263 ± 0.029, 0.665 ± 0.053, 0.636 ± 0.034, and the 10% ABS was 6.13 ± 1.82%, 4.78 ± 1.15%, 12.57 ± 0.36%, 12.07 ± 0.46% in the duodenum, jejunum, ileum and colon, respectively. Compared with the individual compound, the P*_eff_ and the 10% ABS of paeoniflorin were significantly increased by 2.11-, 1.40-fold in ileum and by 1.90-, 1.25-fold in colon, respectively. While paeoniflorin was co-administrated with SSD, the P*_eff_ of paeoniflorin in the duodenum, jejunum, ileum and colon was 0.304 ± 0.048, 0.280 ± 0.051, 0.731 ± 0.052, 0.696 ± 0.054, and the 10% ABS was 6.68 ± 1.91%, 5.77 ± 1.39%, 13.75 ± 0.99%, 13.48 ± 0.57%, respectively. From Figure 2, SSA and SSD can enhance the permeability of paeoniflorin in the four intestinal segments. However, there was no significant increase for the permeability of paeoniflorin at jejunum and duodenum. Comparatively speaking, the P*_eff_ and the 10% ABS of paeoniflorin were also significantly enhanced by 2.42-, 1.63-fold in ileum and by 2.18-, 1.51-fold in colon after co-administration with SSD. This phenomenon evidently indicated that SSA and SSD could significantly improve the absorption of paeoniflorin in the ileum and colon.

Here, we summarize the possible mechanism of the improved intestinal absorption of paeoniflorin by co-administration with SSA and SSD. Firstly, it has been reported that some surfactants such as Tween-80 can open the tight junction of intestinal epithelial cells to promote the absorption of drugs [20]. As saikosaponin is a kind of saponin which has surfactant activity, thus it seems that the improved intestinal absorption of paeoniflorin could be attributed to saikosaponin which might increase the intestinal epithelial permeability by opening the tight junction of intestinal epithelial cells. In our previous research, we have studied the interaction between saikosaponin and paeoniflorin by the Caco-2 cell culture model [21]. The change of the electric resistance values has been investigated in before-and-after experiments. It was found that the electric resistance values of Caco-2 cell membranes showed no significant change after the individual paeoniflorin was transported. In contrast, when paeoniflorin was co-administered with saikosaponins, the electric resistance values were significantly decreased, which indicated that saikosaponins could open the tight junction of Caco-2 cell. This result confirmed our inference. Secondly, Liu *et al*. [12] has proven that poor permeation, p-gp-mediated efflux, and hydrolysis via a glucosidase contributed to the poor bioavailability of paeoniflorin and inhibitors of P-glycoprotein can enhance the oral bioavailability and intestinal permeability. We think these mechanisms might be involved in the effect of saikosaponins on paeoniflorin's intestinal permeability.

**Table 3:** 10% ABS of paeoniflorin co-administration with SSA and SSD in different intestinal segments (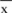 ± s, n = 4).

Compound	Co-administration	10 cm % ABS			
		Duodenum	Jejunum	Ileum	Colon
Paeoniflorin	SSA	6.13 ± 1.82	4.78 ± 1.15	12.57 ± 0.36 *	12.07 ± 0.46 *
	SSD	6.68 ± 1.91	5.77 ± 1.39	13.75 ± 0.99 *	13.48 ± 0.57 *

*P* < 0.05 *vs.* paeoniflorin alone.

#### 2.2.2. Effect of Paeoniflorin on the Absorption of SSA or SSD

**Figure 3:**
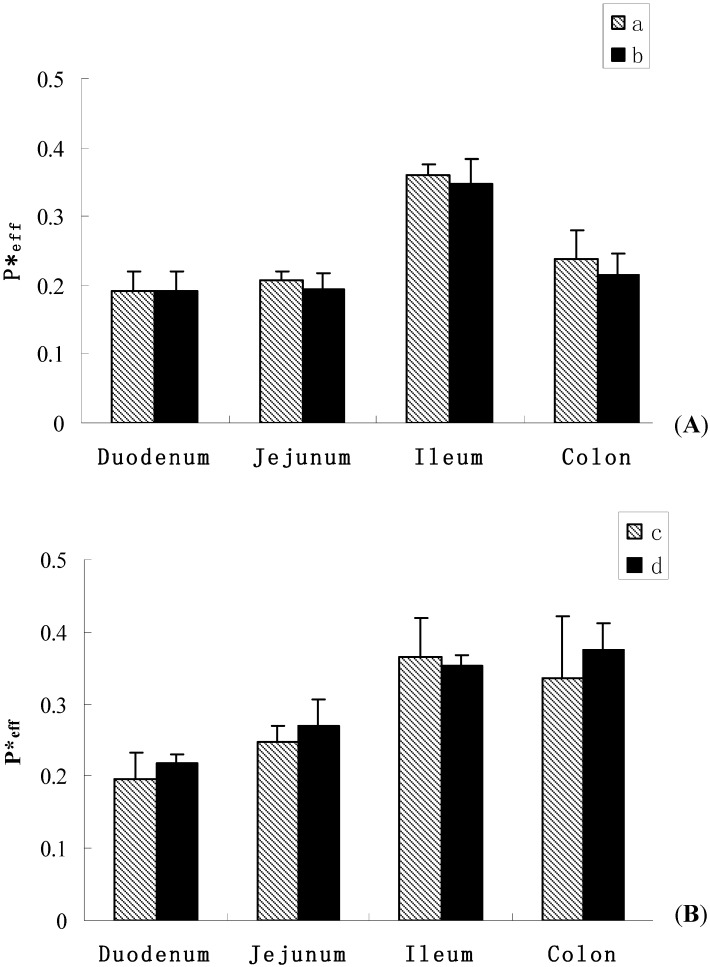
Effective permeability of SSA and SSD in different intestinal segments (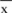 ± s, n = 4). Figure (**A**) SSA; (**a**) alone; (**b**) co-administration with paeoniflorin; Figure (**B**) SSD; (**c**) alone; (**d**) co-administration with paeoniflorin.

On the other hand, we studied the influence of paeoniflorin on SSA and SSD. The results are shown in Figure 3 and Table 4. When co-administrated with paeoniflorin (20:20 μmol·L^−1^), the P*_eff_ of SSA was 0.193 ± 0.028, 0.194 ± 0.023, 0.347 ± 0.036, 0.215 ± 0.030, and the 10% ABS was 4.11 ± 0.30%, 3.49 ± 0.61%, 5.20 ± 0.67%, 3.51 ± 0.73% in the duodenum, jejunum, ileum and colon, respectively. Meanwhile, when SSD was combined with paeoniflorin, the P*_eff_ of SSD was 0.217 ± 0.013, 0.270 ± 0.036, 0.354 ± 0.013, 0.374 ± 0.038, and the 10% ABS was 4.64 ± 0.24%, 5.50 ± 0.73%, 5.97 ± 1.25%, 6.42 ± 0.91% in the duodenum, jejunum, ileum and colon, respectively. These results indicated that there was no significant difference between the absorption of saikosaponins as individual compound after co-administration with paeoniflorin, which suggested that the absorptions of SSA and SSD could not be influenced by paeoniflorin.

**Table 4:** 10 cm % ABS of paeoniflorin co-administration with SSA and SSD in different intestinal segments (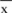 ± s, n = 4).

Compound	Co-administration	10 cm % ABS			
		Duodenum	Jejunum	Ileum	Colon
SSA	Paeoniflorin	4.11 ± 0.30	3.49 ± 0.61	5.20 ± 0.67	3.51 ± 0.73
SSD		4.64 ± 0.24	5.50 ± 0.73	5.97 ± 1.25	6.42 ± 0.91

## 3. Experimental

### 3.1. Chemicals

SSA, SSD and paeoniflorin were purchased from the National Institute for the Control of Pharmaceutical and Biological Products (China). Hanks’ balanced salt solution (HBSS; powder form) was provided by Sigma-Aldrich (Shanghai Trading Co. Ltd.). Testosterone was obtained from Xianju Chemicals Reagent Co. Ltd. (Zhejiang, China). Chrysin was purchased from Shanghai Yuanye Biologocal Technology Co. Ltd. (Shanghai, China). Acetonitrile (German Merck) and water was HPLC grade. All other materials (typically analytical grade or better) were used as received.

### 3.2. Animals

Male Sprague-Dawley rats weighing between 250 and 300 g were obtained from the SLEK Lab Animal Center of Shanghai (Shanghai, China). The rats were fed with water and a standard diet. The rats were fasted overnight before the day of the experiment.

### 3.3. Animal Surgery

The perfusion protocol was approved by the Animal Ethics Committee of Jiangsu Provincial Academy of Chinese Medicine. The intestinal surgical procedures were described in our previous publications [22]. Briefly, rats were anesthetized with an intra-muscular injection of urethane (0.5 g mL^−1^) and placed on a heated surface maintained at its normal body temperature. Four segments of intestine, duodenum, jejunum, ileum, and colon (8–12 cm) were simultaneously cannulated with no disruption of circulation to the intestine, each with two cannulae. After cannulation, the small intestinal segments were placed carefully into the abdominal cavity, avoiding crimping or kinking of the segments. To keep the temperature of the perfusate constant, the inlet cannulate was kept warm by a 37 °C circulating water bath.

### 3.4. Absorption Study in Perfused Rat Intestinal Model

This is a single-pass perfusion method. Four segments of the intestine (duodenum, upper jejunum, terminal ileum, and colon) were perfused simultaneously with a perfusate containing the compound of interest using an infusion pump (Harvard Apparatus, Cambridge, MA, USA) at a flow rate of 0.2 mL min^−1^. After a 30-min washout period, which is usually sufficient to achieve the steady-state absorption, four samples were collected from the outlet cannulae every 30 min afterward. After perfusion, the length of the intestine was measured as described previously [23]. The outlet concentrations of the test compounds in the luminal perfusate were determined by HPLC or LC/MS.

### 3.5. LC/MS Analysis of Perfusion Samples

The outlet concentrations of SSA and SSD were determined by LC/MS using a HPLC-Agilent 6410 Triple Quad (Agilent, Palo Alto, CA, USA) system equipped with an atmospheric-pressure chemical ionization source. The samples were separated on a Zorbax SB-C18 column (250 mm × 4.6 mm, 5 μm, Agilent) and Zorbax SB-C18 precolumn (4.6 × 12.5 mm, 5 mm, Agilent). The mobile phase consisted of acetonitrile and water (40:60, v/v), and the flow rate was 0.4 mL·min^−1^. The column temperature was maintained at 35 °C and the injection volume was 20 μL. For MS analysis, atmospheric-pressure chemical ionization (APCI) source was operated in the negative ion mode. The selected parameters were as follows: drying nitrogen gas at 350 °C and a flow-rate of 4 mL·min^−1^; nebulizer pressure at 20 p.s.i.g. (1 p.s.i. = 6894.76 Pa); vaporizer temperature at 325 °C; capillary voltage at 4,500 V and corona current at 4 μA. The chromatograms obtained for quantitation were recorded under time-scheduled SIM, with fragmentor voltages was set at 150 V. The scan range was from *m/z* 250 to *m/z* 1,000. The instrument was programmed for a scan dwell time of 200 ms. Intense [M+H]^−^ ions at *m/z* 781 and *m/z* 255 were shown for SSA (SSD) and chrysin(internal standard). In general, these methods were selective and reproducible with day to day variability of less than 3%. The tested linear response range for SSA and SSD was 0.625 to 80 μM. The accuracy and precision were greater than 98%. The recovery of three different concentrations was around 90.91%~92.66%, and RSD was less than 5% (n = 5).

### 3.6. UPLC Analysis of Perfusion Samples

UPLC was used to analyze the concentration of paeoniflorin in the perfusate samples. The conditions were as follows: Waters Acquity™ system, with diode array detector and Empower software; column, Acquity UPLC BEH C-18 1.7 μm, 50 × 2.1 mm, (Waters, Milford, MA, USA); mobile phase A, water; mobile phase B, acetonitrile; gradient, 0~0.8 min, 15~22% B, 0.8~1.0 min, 22% B, 1.0~1.5 min, 35% B, 1.5~2.2 min, 70% B; flow rate, 0.4 mL·min^−1^; wavelength 230 nm (paeoniflorin) and 245 nm (testosterone, the internal standard); column temperature, 35 °C; injection volume, 10 μL. In general, these methods were selective and reproducible with day to day variability of less than 3%. The tested linear response range for paeoniflorin was 0.625 to 120 μM. The accuracy and precision were greater than 98%. The recovery of three different concentrations was around 99.73%~100.81%, and RSD was less than 5% (n = 5).

### 3.7. Data Analysis

In the perfused rat intestinal model, *P*_eff_* (effective permeability) is a representation of the intestinal membrane permeability. *P*_eff_* of the compounds are calculated using the following equation:

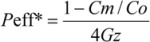


Where *Co* and *Cm* are inlet and outlet concentrations, respectively; *Gz*, or Graetz number (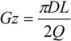), is a scaling factor that incorporates flow rate (Q), intestinal length (L), and diffusion coefficients (D) to make the permeability dimensionless. *Cm* was adjusted for water flux, data points were discarded if the water flux exceeded 0.5% cm^−1^ of intestine.

## 4. Conclusions

In the present study, the absorption and interactions of the main components in the Chaihu-Shaoyao drug pair were investigated for the first time by using the rat intestinal perfusion model. We found that the intestinal absorptions of both the active components in Chaihu and Shaoyao were poor, however, when they co-administrated, SSA and SSD could significantly reinforce the absorption of paeoniflorin. It is clearly demonstrated for the first time that these three compounds have a synergistic reaction in the intestine, which reveals the compatibility mechanism of TCMs to some extent.
